# Mixed-Cation Halide Perovskite Doped with Rb^+^ for Highly Efficient Photodetector

**DOI:** 10.3390/ma16103796

**Published:** 2023-05-17

**Authors:** Wei Wu, Yang Liu, Jianxi Yao, Xiaoping Ouyang

**Affiliations:** 1 School of Nuclear Science and Engineering, North China Electric Power University, Beijing 102206, China; 2 State Key Laboratory of Nuclear Resources and Environment, East China University of Technology, Nanchang 330013, China; 3 School of Renewable Energy, North China Electric Power University, Beijing 102206, China; 4 State Key Laboratory of Intense Pulsed Radiation Simulation and Effect, Xi’an 710024, China

**Keywords:** mixed-cation halide perovskite, photodetector, broad spectrum

## Abstract

Photodetectors are widely employed as fundamental devices in optical communication, automatic control, image sensors, night vision, missile guidance, and many other industrial or military fields. Mixed-cation perovskites have emerged as promising optoelectronic materials for application in photodetectors due to their superior compositional flexibility and photovoltaic performance. However, their application involves obstacles such as phase segregation and poor-quality crystallization, which introduce defects in perovskite films and adversely affect devices’ optoelectronic performance. The application prospects of mixed-cation perovskite technology are significantly constrained by these challenges. Therefore, it is necessary to investigate strategies that combine crystallinity control and defect passivation to obtain high-quality thin films. In this study, we incorporated different Rb^+^ ratios in triple-cation (CsMAFA) perovskite precursor solutions and studied their effects on crystal growth. Our results show that a small amount of Rb^+^ was enough to induce the crystallization of the α-FAPbI_3_ phase and suppress the formation of the yellow non-photoactive phase; the grain size increased, and the product of the carrier mobility and the lifetime (*μτ*) improved. As a result, the fabricated photodetector exhibited a broad photo-response region, from ultraviolet to near-infrared, with maximum responsivity (*R*) up to 11.8 mA W^−1^ and excellent detectivity (D*) values up to 5.33 × 10^11^ Jones. This work provides a feasible strategy to improve photodetectors’ performance via additive engineering.

## 1. Introduction

In the past decade, solution-processed organic–inorganic hybrid perovskites (OIHPs), which can be prepared by a simple and low-cost spin coating method, have garnered widespread attention in the field of optoelectronics due to their superior properties, such as high absorption coefficient, high carrier mobility, low exciton binding energy, and adjustable band gap [1,2,3]. In general, perovskites can be classified as organic or inorganic according to their chemical compositions, which lead to quite different properties in materials. All-inorganic perovskites have the general chemical formula AMX_3_, and the A-site cation is generally Cs^+^. The family of all-inorganic perovskites has fewer members owing to the limitations of the tolerance factor and octahedral factor [4]. Compared to all-inorganic perovskites, OIHPs are usually composed of A-site organic cations and inorganic lead halide octahedra [PbX_6_]^4−^. Numerous chemically versatile multidimensional OIHP structures can be obtained by modifying the A-site organic cation component due to the wide variety of organic cation groups. OIHPs can adapt to various optoelectronic applications such as solar cells, photodetectors, and light-emitting diodes [4,5,6,7]. OIHPs have undergone an incredible evolution in the solar cell field. The power-conversion efficiency of perovskite solar cells jumped rapidly from 3.8% to 25.7% within just over a decade [8,9]. In particular, methylammonium lead triiodide (MAPbI_3_) has been extensively investigated and exhibits outstanding photoelectric conversion efficiency as a potential absorber in solar cells [10,11,12]. However, the fragmentation of the MA ion occurs when temperature exceeds 100 °C, leading to the rapid decomposition of the MAPbI_3_ crystal structure [13]. Moreover, MAPbI_3_ has a tetragonal rather than cubic structure at room temperature, with a band gap of 1.51 eV due to the small size of the MA ion [14]. Compared to MAPbI_3_, formamidinium lead iodide (FAPbI_3_) possesses higher symmetry and is closer to a cubic structure [4]. It is well known that crystals closer to a perfect symmetric cubic structure have minimal lattice distortion, which is beneficial for carrier transport [15,16]. Furthermore, FAPbI_3_ exhibits superior thermal stability and a favorable band gap closer to the optimal band (1.4 eV) [17]. The reduced band gap may allow the absorption of photons over a broader spectrum, rendering FAPbI_3_ one of the most promising absorbing layers for high-performance photodetectors [18,19,20]. Generally, FAPbI_3_ perovskite has only two phases at room temperature: one is a photoactive cubic phase (α-phase), and the other is a non-photoactive hexagonal phase (δ-phase) [15]. Unfortunately, the α-phase is unstable and easily collapses to the δ-phase at room temperature, limiting its further application. Hence, preparing FAPbI_3_ without the δ-phase and stabilizing the α-phase have become urgent problems. In particular, mixed-cation engineering has been demonstrated as an effective strategy for improving perovskites’ phase stability. Jeon et al. proved that a small amount of MA^+^ is enough to induce improved crystallization of black α-FAPbI_3_ [21]. Furthermore, Saliba et al. showed that the incorporation of Cs^+^ can effectively suppress the formation of yellow-phase impurities in MA/FA perovskite compounds; a device prepared based on a Cs/MA/FA triple-cation yielded stabilized power-conversion efficiency exceeding 21% [22]. Nevertheless, mixed-cation perovskites still face the obstacle of performance degradation under continuous illumination, which mainly stems from a special process referred to as halide segregation [23,24,25]. Photon irradiation above the band gap induces the separation of halide ions (I^−^ and Br^−^) to form different halide compositions, which incorporate various impurities and defects into the film [26]. It is well known that defects, such as pinholes, cracks, and grain boundaries, function as non-radiative recombination centers, decreasing carriers’ lifetimes and adversely affecting devices’ performances [27]. To address this issue, chemical doping engineering has been demonstrated to be effective in passivating defects and improving the phase stability of mixed-cation perovskites [28]. For example, Khan et al. reported a method of doping the small molecule F4TCNQ into the triple-cation perovskite to improve a film’s crystallization process [29]. F4TCNQ promoted the crystallization of the photoactive phase as the grain size increased and grain boundaries decreased. Hence, the photodetector exhibited excellent performance, including a high on–off ratio, high responsivity, and low dark current. Furthermore, stoichiometry engineering has been used to limit the extent of halide segregation. Particularly, changing the A-site composition can alter the degree of octahedral distortion or induce other structural changes that affect the optoelectronic properties of perovskite materials. Numerous previous studies have demonstrated that the optimal stoichiometric ratio corresponding to the suppression of phase segregation can be obtained by carefully adjusting the composition of A-site cations [30,31]. Recently, incorporating small-radius Cs^+^ or Rb^+^ in triple-cation perovskites has been proven to alleviate lattice strain, which is beneficial for suppressing the formation of FA^+^ and I^−^ vacancies [32,33]. Moreover, Kubicki et al. demonstrated via solid-state NMR spectroscopy that Rb^+^ does not dope the lattice of Cs/MA/FA perovskite, avoiding the introduction of extra lattice strains. Instead, Rb^+^ may form various Rb-containing compounds with excess PbI_2_ and can act as a potential passivation layer, which is beneficial for decreasing non-radiative recombination and improving carrier transport [28]. These findings have paved the way for the design and creation of superior and stable photodetectors.

In this study, we incorporated different ratios of Rb^+^ into the triple-cation perovskite precursor solution Cs_0.05_(MA_0.17_FA_0.83_)_0.95_Pb(I_0.83_Br_0.17_)_3_ (Cs_0.05_(MA_0.17_FA_0.83_)_0.95_ is abbreviated as CsMAFA in this paper). We demonstrated via X-ray diffraction and scanning electron microscopy that the optimal ratio of Rb^+^ can improve crystallinity and suppress the formation of non-photoactive phases. Incorporating Rb^+^ increased the grain size and reduced grain boundaries, which contributed to the inhibition of non-radiative complexation and improved carrier mobility. Therefore, the fabricated photodetector exhibited a broad photo-response region from ultraviolet (UV) to near-infrared (NIR) with a maximum responsivity of up to 11.8 mA W^−1^ and an excellent D* value of up to 5.33 × 10^11^ Jones. The maximum switching ratio was 7252, and the rise and decay times were 208.3 μs and 288.4 μs, respectively. During measurement under ambient light, no significant photocurrent decays were found during multiple cycles.

## 2. Materials and Methods

### 2.1. Materials

Formamidinium iodide (FAI, 99.0%, Acros, Geel, Belgium), cesium iodide (CsI, 99.0%, Acros, Geel, Belgium), methylammonium bromide (MABr, 99.0%, Acros, Geel, Belgium), and lead (II) bromide (PbBr_2_, 99.0%, Acros, Geel, Belgium) were purchased from Beijing Innochem Science & Technology Co., Ltd., Beijing, China. Lead (II) iodide (PbI_2_, 99.9%) was purchased from Xi’an Polymer Light Technology Corp., Xi’an, China. Super-dehydrated dimethylformamide (DMF, 99.0%) was purchased from Beijing J&K Scientific Technology Corp., Beijing, China. Dimethyl sulfoxide (DMSO, 99.9%, Alfa, Milan, Italy) was purchased from Alfa Aesar Chemical Corp., Taiwan, China. All chemicals were purchased from commercial suppliers and used without further processing.

### 2.2. Device Fabrication

The glass substrates were cleaned twice with deionized water, and then with ethanol. After each cleaning, the glass substrates were sonicated for 20 min. After sonication, the glass substrates were blown dry with nitrogen, and then a UV–ozone treatment was performed for 30 min. A compact TiO_2_ layer was deposited from a precursor of titanium diisopropoxide (0.6 mL) and bis(acetylacetonate) (0.4 mL) in anhydrous propan-2-ol (7 mL). The precursor solution was sprayed onto the glass substrate, which was subsequently annealed on a hot plate at 460 °C for an hour and then left to cool to room temperature. The Cs/MA/FA perovskite precursor solution was prepared by dissolving 1 M FAI, 1.1 M PbI_2_, 0.2 M MABr, 0.063 M CsI, and 0.22 M PbBr_2_ in anhydrous DMF/DMSO 4:1 (*v*/*v*). Next, the quadruple cation was prepared by adding 0%, 5%, and 10% of the 1.0 M RbI solution to the Cs/MA/FA perovskite precursor solution. The quadruple-cation precursor solution was spin-coated onto the glass/c-TiO_2_ substrate at 1500 rpm for 5 s and then at 4000 rpm for 30 s; the glass/c-TiO_2_/perovskite substrate was subsequently annealed on a hot plate at 100 °C for 60 min. A 60 nm gold interdigitated electrode (distance between electrodes: L = 50 µm) was deposited via thermal evaporation under a pressure of 4 × 10^−4^ Pa. A black mask was pasted on to guarantee a standard illumination area of 5 mm^2^.

### 2.3. Characterization

UV–vis spectra were measured using the UV–2450 (Shimadzu, Kyoto, Japan) from 400 nm to 900 nm. XRD patterns were obtained with an X-ray diffractometer (XRD, Smart-Lab, Rigaku, Tokyo, Japan) using Cu–Kα radiation (1.5418) (5–55°, 4° min^−1^). The morphologies of thin films were measured using a SU8010 SEM (Hitachi, Chiyoda City, Japan, 3.0 kV, 10,100 nA). The photodetector’s performance was measured using an LED light source with different wavelengths. All current data were recorded using an electrochemical workstation with a CIMPS system (Zahner, Kansas City, MO, USA).

## 3. Results and Discussion

**Figure 1:**
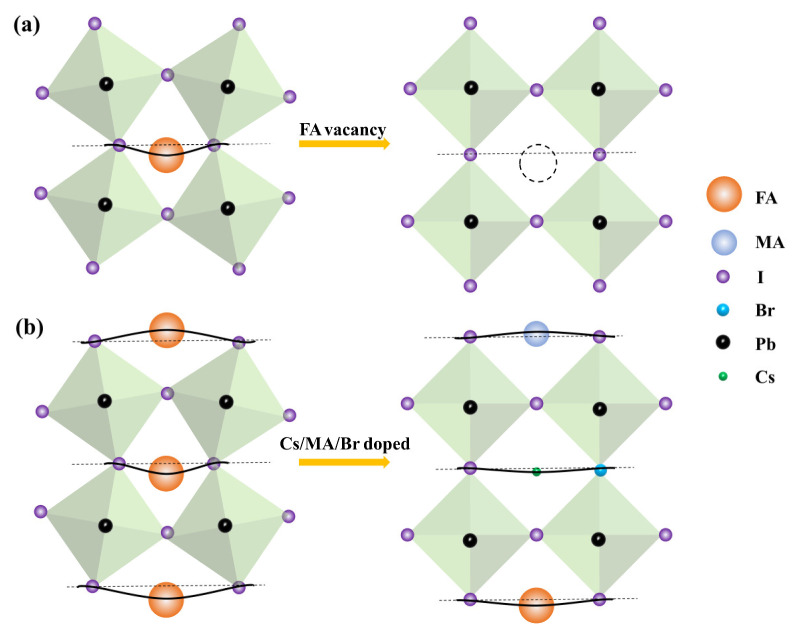
Schematic diagram of lattice strain relief in CsMAFA perovskite: (**a**) formation of FA vacancy; (**b**) incorporation of multiple ions.

Possessing a broad optical absorption spectrum and high absorption coefficient, black-phase FAPbI_3_ (α-FAPbI_3_) perovskites have achieved impressive high-efficiency solar cells (25.7%) and become a popular material in the optoelectronic devices field. However, FAPbI_3_’s octahedral skeleton is unstable owing to the lattice distortion caused by FA ions, which may form vacancies in the perovskite lattice. According to the principle of mechanical force, internal stresses occur and resist this effect when large cations stretch the octahedral skeleton, leading to an unstable state in the whole system [34]. Figure 1a shows the lattice distortion caused by FA ions, and the extent to which the black solid line deviates from the dotted line indicates the intensity of internal stresses. FA ions’ large size tends to aggravate lattice distortion, resulting in the formation of vacancies and adversely affecting photodetectors’ performance [35]. One strategy to alleviate lattice distortion is to insert small-radius ions, such as MA^+^ and Cs^+^, at the A-site [36]. Figure 1b shows the lattice distortion mitigated by the incorporation of small-radius ions such as Cs^+^, MA^+^, and Br^−^ into the FA-based perovskite’s lattice, yielding a higher symmetry.

**Figure 2:**
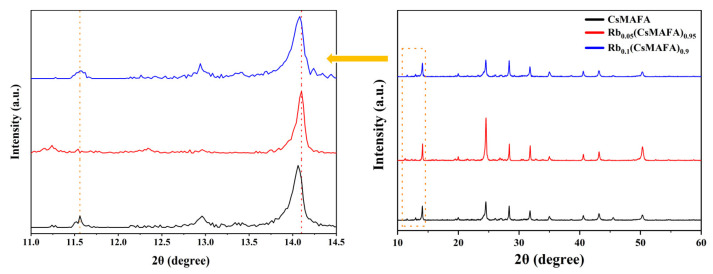
XRD spectra of the Rb_x_(CsMAFA)_1−x_ perovskite with different Rb ratios.

To further investigate the effect of Rb^+^ incorporation on the growth of CsMAFA perovskite films, X-ray diffraction measurements were taken. As shown in Figure 2, all components exhibited a typical perovskite peak near 14°. For the CsMAFA (component without Rb^+^) film, a typical characteristic peak appeared at 14.06°. The main reflection peak of α-FAPbI_3_ (14°) shifted to a higher angle, indicating that the doping of Cs^+^ caused the contraction of the MAFA-based perovskite lattice, which facilitated the release of lattice strain. Moreover, we noted small side peaks at 11.56° and 12.96°, corresponding to non-photoactive (δ-FAPbI_3_) and excess PbI_2_, respectively. This was a typical observation of the incomplete conversion of mixed-cation perovskites to the photoactive phase. Subsequently, when we introduced 5% of Rb^+^, the main reflection peak further shifted to 14.10° and the peak intensity increased, demonstrating that the introduction of Rb^+^ could further alleviate lattice distortion in the CsMAFA-based perovskite. In particular, the intensity of the δ-phase diminished when we introduced Rb^+^, and 5% of Rb^+^ suppressed the formation of the δ-phase most effectively, which indicated that a small amount of Rb^+^ was able to promote the CsMAFA-based perovskite crystallizing to the α-phase. Notably, the decrease in the small side peak at 12.96° may suggest that Rb^+^ consumed the residual PbI_2_ reaction to form the Rb-containing mixture [28]. Moreover, excessive Rb^+^ may compete with the incorporation of Cs^+^ into the perovskite lattice by forming a stable mixed Cs/Rb hexagonal phase.

The main reflection peak shifted slightly to 14.08° and the diffraction peak’s intensity decreased when the Rb^+^ concentration was increased to 10%. Therefore, excessive Rb+ may be detrimental to the crystallization of CsMAFA-based perovskite. In terms of crystal quality, the optimal ration of Rb^+^ was 5%.

**Figure 3:**
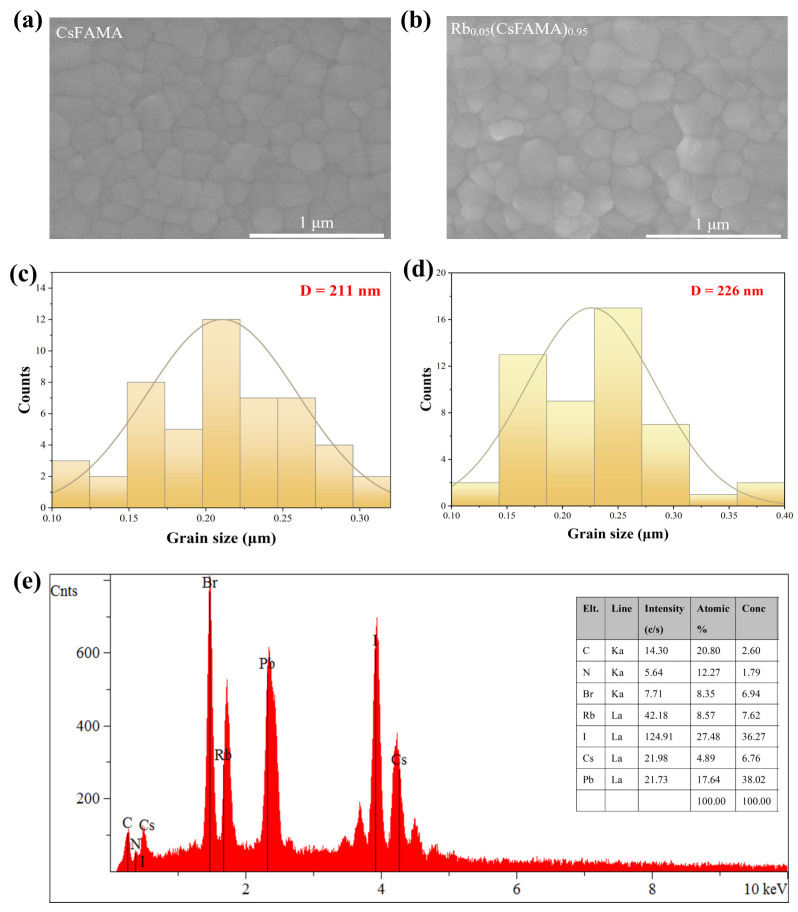
Scanning electron microscope images of the mixed-cation Rb_x_(CsMAFA)_(1−x)_ films incorporated with (**a**) 0% Rb^+^ and (**b**) 5% Rb^+^. Grain size statistics for Rb_x_(CsMAFA)_(1−x)_ films incorporated with (**c**) 0% Rb^+^ and (**d**) 5% Rb^+^. (**e**) EDS on the surface of the Rb_0.05_(CsMAFA)_0.95_ thin film showing the elemental composition.

It is well known that films with a large grain size and fewer grain boundaries are essential for reducing carrier recombination and enhancing carrier mobility, as pinholes or cracks tend to form non-radiative recombination centers that adversely affect devices’ performance. The surface morphologies of CsMAFA and Rb_0.05_(CsMAFA)_0.95_ were investigated using scanning electron microscopy (SEM) (Figure 3a,b). As shown in Figure 3c,d, the average grain size of the Rb_0.05_(CsMAFA)_0.95_ film improved from 211 nm to 226 nm, which indicates that Rb^+^ promoted crystallization and matched the XRD result. Energy Dispersive Spectroscopy (EDS) on the surface of the Rb _0.05_(CsMAFA)_0.95_ film also confirmed the presence of Rb^+^, as shown in Figure 3e. Hence, the addition of an optimal proportion of Rb^+^ to the perovskite precursor solution could effectively control the crystallization process of the film, which was beneficial for yielding highly crystalline and crack-free perovskite films to improve photodetectors’ performance.

**Figure 4:**
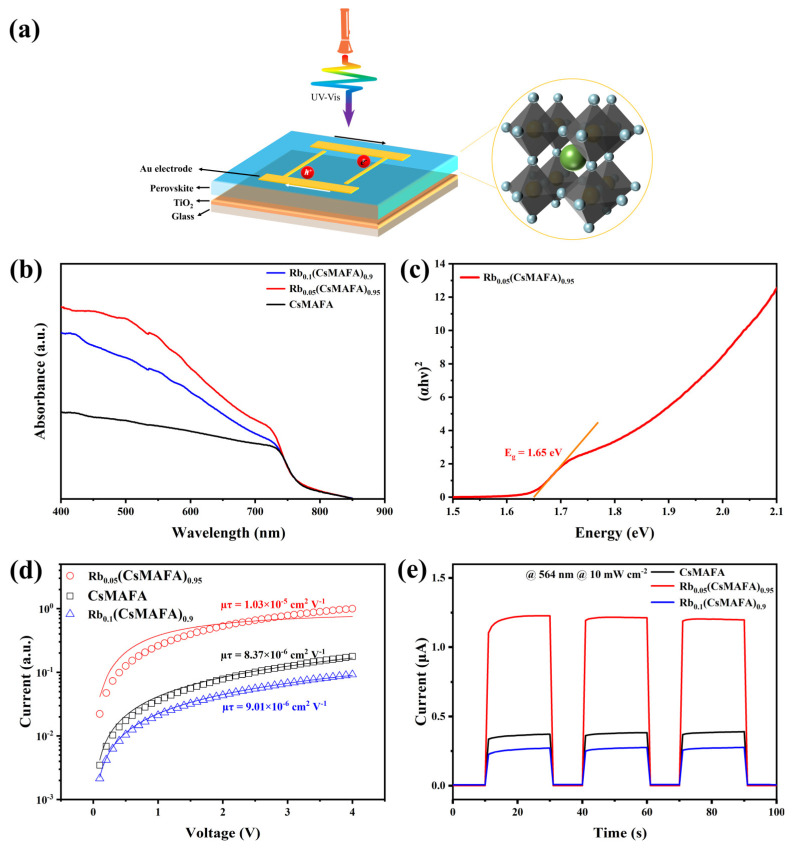
(**a**) Schematic diagram of photodetector’s structure; (**b**) UV–vis absorption spectra of Rb_x_(CsMAFA)_(1−x)_ perovskite thin films; (**c**) the calculated optical band gap based on the Tauc equation; (**d**) photoconductivity measurement of the devices based on Rb_x_(CsMAFA)_(1−x)_ perovskite thin films; (**e**) the photodetectors’ switching properties under a 564 nm and 10 mW cm^−2^ light illumination.

Figure 4b shows the UV–Vis absorption spectra of CsMAFA perovskite films with different Rb^+^ ratios. The CsMAFA film has a strong absorbance in the region of wavelengths less than 450 nm, and the absorption band could extend beyond 800 nm. The Rb_0.05_(CsMAFA)_0.95_ film’s absorbance significantly improved in the region of wavelengths less than 752 nm, with a band gap of 1.65 eV calculated using the Tauc formula (Figure 4c). The absorbance increase stemmed from the increase in grain size and the reduction in grain boundaries and cracks. It was shown that the optimal ratio of Rb^+^ helped induce crystallization of the photoactive phase and improve the film quality, which was beneficial for enhancing the device’s charge collection efficiency. Increasing charge collection efficiency enables detectors to output larger light-to-dark ratios.

**Figure 5:**
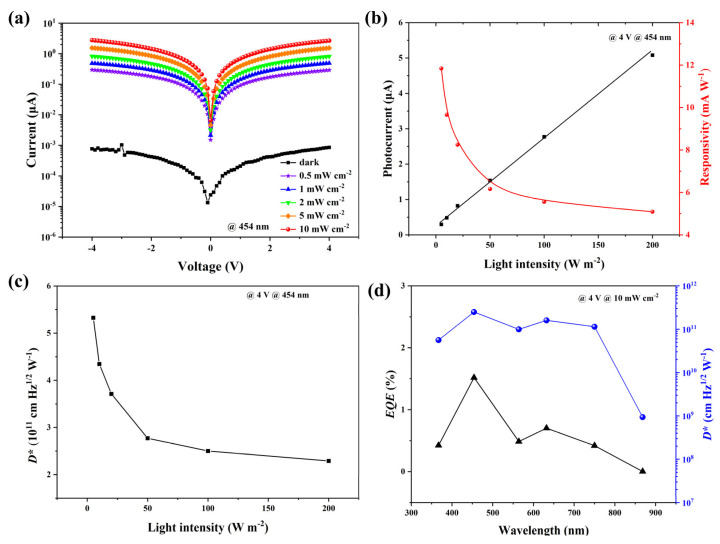
(**a**) I–V curves of the Rb_0.05_(CsMAFA)_0.95_ perovskite photodetector under 454 nm light illumination with different illumination intensities. (**b**) Photocurrent and responsivity versus light intensity at 454 nm light illumination under 4 V. (**c**) D* versus light intensity at 454 nm light illumination under 4 V. (**d**) EQE and D* versus wavelength at 10 mW cm^−2^ under 4 V.

Detectors’ charge collection efficiencies can be evaluated using the carrier mobility–lifetime (*μτ*) product. To quantitatively characterize the effect of different Rb^+^ ratios on the detector’s charge collection efficiency, we fabricated three devices separately and performed photoconductivity measurements. In this paper, we used a gold interdigitated electrode to collect carriers, as shown in Figure 4a. Alternating arc-shaped electric fields are formed between interdigitated electrodes at each end, and carriers are transported between interdigitated electrodes at the top of the perovskite layer. Electrons and holes may bounce between two electrodes repeatedly, resulting in recirculation and abnormally high photoconductive gain. As shown in Figure 4d, we used a modified Hecht equation to fit the photoconductivity curves and extract the *µτ* values. The Hecht equation is expressed as follows [37]:

(1) $$I=\frac{I_{0}\mu \tau V}{d^{2}}\left(1-exp\left(-\frac{d^{2}}{\mu \tau V}\right)\right)$$

where *I*_0_ is the saturated photocurrent and *d* is the distance between electrodes [38]. The calculated *µτ* values of CsMAFA, Rb_0.05_(CsMAFA)_0.95_, and Rb_0.1_(CsMAFA)_0.9_ were 8.37 × 10^−6^ cm^2^ V^−1^, 1.03 × 10^−5^ cm^2^ V^−1^, and 9.01 × 10^−6^ cm^2^ V^−1^, respectively. The Rb_0.05_(CsMAFA)_0.95_ device yielded the highest *µτ* value, further demonstrating the beneficial effect of small amounts of Rb^+^ on the crystallization of films. Figure 4e shows the photocurrent responses of three photodetectors under a light illumination of 10 mW cm^−2^ and a bias of 4 V. The calculated on–off values for CsMAFA, Rb_0.05_(CsMAFA)_0.95_, and Rb_0.1_(CsMAFA)_0.9_ were 64, 1519, and 37, respectively. Notably, the photocurrent of the Rb_0.1_(CsMAFA)_0.9_ device decreased instead, which could probably be attributed to cesium’s tendency to form a stable Cs_0.5_Rb_0.5_PbI_3_ phase in the presence of excess rubidium [28]. As a result, an optimal amount of Rb^+^ could yield the highest switching ratio. Based on the above analysis, we determined that the optimal Rb^+^ ratio was 5% and measured the current–voltage (I–V) characteristics (Figure 5a) of the prepared photodetector under different light irradiation wavelengths. The typical ohmic characteristics of the I–V curves indicated that a good ohmic contact was formed between the perovskite and the Au electrode. The increase in conductivity under light irradiation was attributed to an increase in the electron–hole pair concentration; the current signal generated by an external voltage was detectable. Responsivity (*R*) is an important parameter characterizing the ability of a photodetector to convert photons into a current signal, and it can be calculated using the following equation [39]:

(2) $$R=\frac{I_{p}-I_{d}}{PS}$$

where *I_p_* is the photocurrent, *I_d_* is the dark current, *P* is the light intensity, and *S* is the effective area of the device. Figure 5b shows variations in *R* versus light intensity. The photocurrent increased linearly with increasing light intensity under 454 nm illumination, whereas the responsivity decreased with increasing light intensity due to the dominance of bimolecular recombination under strong light [40]. The maximum *R* was 11.8 mA W^−1^ at a light intensity of 0.5 mW cm^−2^. Linear dynamic range (LDR) is a figure-of-merit that represents the range of light intensity over which a detector can output photocurrent linearly. LDR can be calculated using the following formula [41]:

(3) $$LDR=20lg\frac{P_{max}}{P_{min}}$$

where *P_max_* is the saturation light intensity under which the photocurrent begins to deviate from linearity and *P_min_* is the initial light intensity. Figure 5b shows that the photocurrent increased linearly with increasing light intensity from approximately 0.5 mW cm^−2^ to above 20 mW cm^−2^, yielding an LDR of 32 dB. We further evaluated the specific detectivity, which is a parameter characterizing a detector’s ability to detect weak light and is calculated using the following expression:

(4) $$D^{*}=\frac{R}{{\left(2eI_{d}/S\right)}^{0.5}}$$

where *R* is the responsivity, *e* is the unit charge, *I_d_* is the dark current, and *S* is the active area. Figure 5c shows the variation in D* with light intensity. The maximum value of D* was 5.33 × 10^11^ Jones under light irradiation at 454 nm, 0.5 mW cm^−2^, which proves its potential application in weak near-ultraviolet light sensing and imaging. External quantum efficiency (*EQE*) is also a key photodetector parameter and is defined as the ratio of the number of electron–hole pairs collected by the external circuit to the number of incident photons. It is calculated as follows:

(5) $$EQE=\frac{Rhc}{e\lambda }$$

where *R* is the responsivity, *h* is Planck’s constant, *c* is the speed of light, *e* is the elementary charge, and *λ* is the wavelength of the incident light. The *EQE* versus wavelength is shown in Figure 5d. The *EQE* reached a peak of 1.5% at a wavelength of 454 nm, with a D* up to 2.49 × 10^11^ Jones due to the high absorption coefficient in the near-UV band. The *EQE* gradually decreased in the region of wavelengths above 632 nm; the lowest D* was 9.38 × 10^8^ Jones at 868 nm.

**Figure 6:**
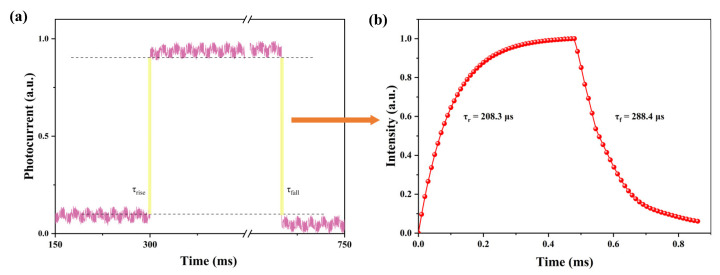
(**a**) Photocurrent response of the photodetector with rising and decaying time. (**b**) The fitted curve of the rising and decay processes.

Moreover, response time is a key parameter that represents the speed of detecting transient light signals. Rise time is the time duration from 10% to 90% of the saturation current. As shown in Figure 6a,b, we fitted the experimental curves of the rising and decay processes using a stretched exponential function [42]:

(6) $$I=I_{0}-I_{0}\exp\left(-\frac{t}{\tau }\right)$$

where *I*_0_ is the amplitude of the current and *τ* is the time constant. The calculated rise time (*τ_r_*) and fall time (*τ_f_*) were 208.3 μs and 288.4 μs, respectively, which demonstrated the effective extraction of carriers at the interface between the perovskite and Au electrodes and the perovskite thin film’s high charge mobility. The quadruple-cation perovskite films’ ultra-fast response time and broad absorption spectrum from the ultraviolet to the near-infrared demonstrated their promising application as broadband photodetectors. A summary of the state-of-the-art research regarding perovskite photodetectors’ performance is presented in Table 1.

**Figure 7:**
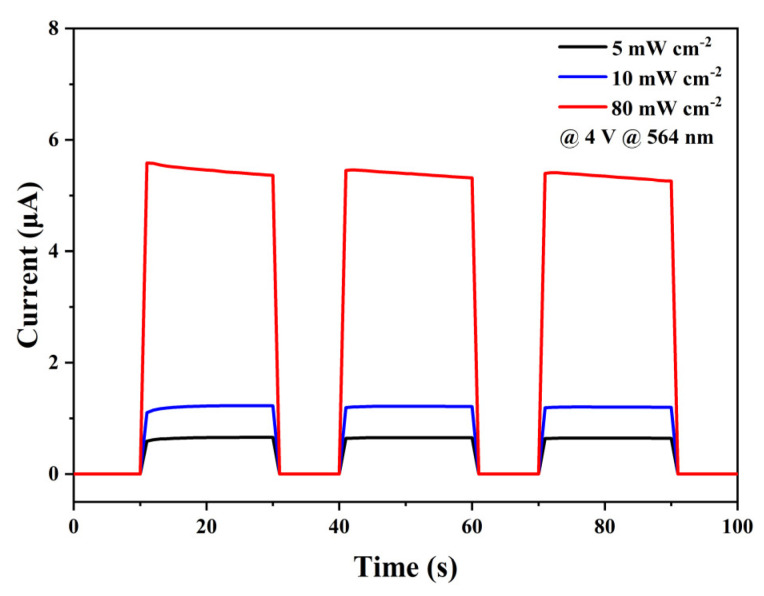
Switching behavior of the photodetector based on Rb_0.05_(CsMAFA)_0.95_ quadruple-cation perovskite with different light intensities.

The repeatability of photodetectors in detecting light is an important factor limiting their practical application. It is well known that detectors based on mixed-cation perovskite thin films suffer from severe baseline drift during continuous on–off due to ion migration and phase segregation. As shown in Figure 7, we measured the photodetector’s switching behavior under 564 nm light with a 4 V bias. The device showed no significant photocurrent decay during multiple on–off cycles with a maximum on–off ratio up to 7252 at 80 mW cm^−2^ light illumination; this demonstrated good photosensitive stability under ambient conditions.

## 4. Conclusions

In conclusion, we developed a photodetector based on a mixed-cation perovskite thin film. The incorporation of an optimal Rb^+^ proportion into the precursor solution helped obtain highly crystalline perovskite films with large grain sizes and few grain boundaries. A reduction in nonradiative or undesired recombination improved the carrier mobility–lifetime product (*μτ*). Hence, the photodetector based on mixed-cation perovskite (Rb_0.05_(CsMAFA)_0.95_) thin film exhibited a good linear response over a light intensity range of 0.5 mW cm^−2^ to 20 mW cm^−2^ with a maximum responsivity of 11.8 mA W^−1^ and an excellent on–off ratio of up to 7252. Simultaneously, the photodetector showed a broad absorption spectrum between 400 and 765 nm, with a maximum D* of 5.33 × 10^11^ Jones. The effective extraction of carriers at the interface between the perovskite and Au electrodes facilitated the device’s ultra-fast response time; the *τ_r_* and the *τ_f_* were 208.3 μs and 288.4 μs, respectively, with no significant decrease in photocurrent during multiple cycle measurements. These results verify perovskite’s potential as a next-generation high-performance photodetector.

## Figures and Tables

**Table 1:** Summary of reported perovskite photodetectors.

Material	Spectrum Range (nm)	*EQE* (%)	*R* (mA W^−1^)	D* (Jones)	*τ_r_*/*τ_f_* (ms)	Ref.
Graphene	300–6000	N/A	6.1	N/A	N/A	[43]
MoWO_3_/VO_2_/MoS_2_/Si	N/A	N/A	N/A	4.32 × 10^8^	214/226	[44]
CsPbI_3_	300–700	17	6.7	1.57 × 10^8^	292/230	[45]
MAPbI_3_	1530–1565	0.01	0.01	N/A	0.9/0.9	[46]
CsPbBr_3_	300–540	8	0.01	1.68 × 10^9^	0.2/1.2	[47]
CsPbBr_3_	300–550	N/A	7.26	1.7 × 10^11^	10/22	[48]
Cs_3_BiBr_6_	300–486	0.008	0.025	8 × 10^8^	50/60	[49]
Rb_0.05_(CsMAFA)_0.95_	400–752	1.5	11.8	5.33 × 10^11^	0.208/0.288	This study

## Data Availability

Not applicable.
